# Retina-on-a-dish: A translational platform for tauopathy and retinal neurodegeneration

**DOI:** 10.4103/NRR.NRR-D-25-01268

**Published:** 2026-01-27

**Authors:** Chiara D’Antoni, Lorenza Mautone, Ylenia Gigante, Anna Mirone, Silvia Di Angelantonio

**Affiliations:** Department of Physiology and Pharmacology “V. Erspamer” and Center for Research in Neurobiology “Daniel Bovet”, Sapienza University of Rome, Roma, Italy; Center for Life Nano- & Neuro-Science, Istituto Italiano di Tecnologia, Roma, Italy; D-Tails Research srl BC, Rome, Italy

**Figure 1:**
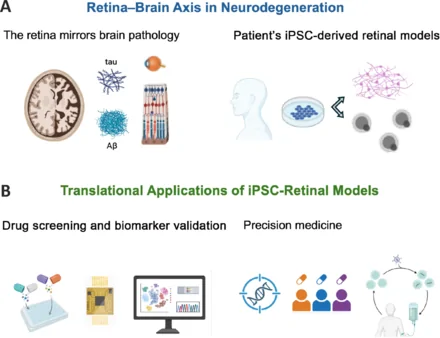
Retina-on-a-dish: a translational platform for tauopathy and retinal neurodegeneration. (A) The retina mirrors brain pathology. Left: Retina–brain axis in neurodegeneration. The retina and brain share common embryological origins and pathological mechanisms, including tau hyperphosphorylation and amyloid-β (Aβ) accumulation. This anatomical and molecular continuity supports the retina’s emerging role as both a disease model and a biomarker source for central nervous system (CNS) tauopathies and Alzheimer’s disease. Right: Patient iPSC-derived retinal models. iPSCs generated from patient somatic cells are differentiated into retinal neurons and 3D retinal organoids. These systems recapitulate key features of tau and amyloid pathology, providing a human-based model to study retinal and CNS degeneration. (B) Translational applications of iPSC-retinal models. Left: Drug screening and biomarker validation. These models serve as platforms for preclinical evaluation of disease-modifying compounds and for identifying retinal biomarkers that may predict or parallel CNS neurodegeneration, with potential for non-invasive monitoring through retinal imaging. Right: Precision medicine. iPSC-derived retinal systems enable personalized modeling of tauopathies and retinal degeneration, incorporating genome editing and isogenic controls to dissect patient-specific disease mechanisms and therapeutic responses. Aβ: Amyloid-β; CNS: central nervous system; iPSC: induced pluripotent stem cell; RO: retinal organoid; RPE: retinal pigment epithelium.

The intersection of retinal and neurodegenerative disease has drawn increasing attention, revealing the retina not only as a passive bystander but also as an active participant in central nervous system (CNS) pathology. Tauopathies such as Alzheimer’s disease (AD) and frontotemporal dementia are characterized by intracellular tau accumulation and synaptic dysfunction—hallmarks that have been observed in the retina of patients and animal models. Amyloid-beta (Aβ) pathology, another defining feature of AD, has similarly been detected in retinal tissues (Gupta et al., 2021; Gaire et al., 2024; Davis et al., 2025). This convergence supports a growing paradigm in which the retina serves as both a surrogate marker and a mechanistic substrate for CNS disease progression. Induced pluripotent stem cell (iPSC)-derived retinal systems, including 2D retinal neurons, 3D retinal organoids, and iPSC-derived retinal pigment epithelium, are redefining how we model neurodegeneration. These human-based platforms offer direct access to disease-relevant phenotypes, genetic precision, and the possibility of patient-specific studies. This article explores how these models illuminate tau-driven pathology, enable mechanistic dissection, and hold promise for therapeutic and biomarker development in neurodegenerative and retinal diseases (**Figure 1**).

**Retina — developmental mirror of the brain:** The retina is a developmental outgrowth of the diencephalon and shares numerous cellular and molecular characteristics with the brain. It is subject to similar pathological stressors, including misfolded protein accumulation, oxidative stress, mitochondrial dysfunction, and neuroinflammation. In AD and frontotemporal dementia, both tau and Aβ aggregates have been observed in retinal ganglion cells, the inner plexiform layer, and the retinal pigmented epithelium (RPE), aligning with histopathological findings in cortical and hippocampal tissue. Non-invasive imaging studies, such as optical coherence tomography, further validate retinal thinning and microvascular abnormalities in preclinical and clinical cohorts of AD. Such parallels elevate the retina as a non-invasive biomarker source and experimental model for CNS neurodegeneration. An additional key advantage of iPSC-derived retinal systems is their potential to significantly reduce reliance on animal models, aligning with the principles of the 3Rs (Replacement, Reduction, and Refinement) and enabling more ethically responsible, human-relevant approaches to modeling neurodegenerative disease.

**Modeling tauopathy in two-dimensional induced pluripotent stem cell–derived retinal neurons:** iPSC-derived 2D retinal neuron cultures have proven effective for modeling early steps in tau pathology. Barolo et al. (2025) demonstrated that exogenous application of pathological tau fibrils to iPSC-derived retinal neurons results in their internalization, cytoplasmic aggregation, and induction of stress pathways, closely recapitulating the prion-like spread of tau seen in the CNS. These cells not only act as recipients but also as propagators of tau species, providing insights into trans-synaptic transmission mechanisms. Functional deficits, including hyperphosphorylation of tau, synaptic mislocalization, and disrupted excitability, were further observed in these retinal and cortical models (Cordella et al., 2025; Mautone et al., 2025). Importantly, iPSC-derived neurons carrying the frontotemporal dementia-associated MAPT IVS10+16 mutation, which alters pre-mRNA splicing to increase the production of 4-repeat tau isoforms implicated in microtubule dysfunction and aggregation, display delayed maturation, persistent expression of progenitor markers, cytoskeletal disorganization, and a pathological predominance of 4-repeat tau, as reported by Mautone et al. (2025). Such findings underscore the capacity of the retina to reveal intrinsic vulnerabilities in a genetically defined human background. Overall, 2D iPSC-derived retinal neurons provide a powerful reductionist model to dissect early tau pathology and propagation mechanisms in a human genetic context.

**Toward complexity — three-dimensional retinal organoids and amyloid pathology:** 3D retinal organoids recapitulate the laminar architecture of the retina and allow modeling of cell-type–specific and layer-specific pathologies. When derived from patients carrying tauopathy-associated mutations, these organoids reveal structural and functional impairments: disrupted stratification, defective photoreceptor maturation, tau aggregation, synaptic degeneration, and heightened sensitivity to oxidative stress. The temporal scalability of organoids facilitates modeling of disease progression — a critical feature absent in short-lived 2D systems. This longitudinal window supports the evaluation of chronic tau accumulation, neurodegenerative changes, and therapeutic intervention efficacy, including biologics and CRISPR (clustered regularly interspaced short palindromic repeats)-based tools.

Retinal organoids have also proven valuable for modeling amyloid pathology in AD. James et al. (2024) showed that retinal organoids derived from familial AD iPSCs exhibit elevated Aβ and phosphorylated tau, mirroring CNS pathology. Exposure of organoids to severe acute respiratory syndrome coronavirus 2 spike protein in the study by Miller et al. (2025) similarly increased Aβ accumulation and gliosis, supporting a model in which viral triggers exacerbate neurodegeneration. This finding was echoed in postmortem retinal explants from coronavirus disease 2019 patients, which revealed amyloid deposits and inflammation.

Together, these findings position 3D retinal organoids as advanced platforms to study layered retinal architecture, progressive tau and amyloid pathology, and the impact of environmental triggers on disease progression.

Conversely, iPSC-derived RPE models offer a complementary lens into neurodegeneration. Li et al. (2024) showed that patient-derived age-related macular degeneration and AD iPSC-derived retinal pigment epithelium exhibit hallmark features of disease: lipid droplet accumulation, sub-RPE drusen-like deposits, mitochondrial dysfunction, and complement activation. These features are challenging to reproduce in animal models and reinforce the value of patient-specific RPE systems. iPSC-derived RPE systems thus complement neuronal and organoid models by capturing disease-relevant phenotypes that are otherwise difficult to reproduce *in vivo*.

**Translational applications — drug discovery, biomarkers, and precision medicine:** Human iPSC-derived retinal models are rapidly emerging as powerful translational platforms. They support high-throughput drug screening of compounds targeting tau aggregation, Aβ toxicity, and RPE dysfunction. Functional outputs, such as protein aggregation, synaptic activity, cell survival, and cytokine release, can be monitored in real time using multi-well imaging and high-content screening systems. Moreover, these platforms enable biomarker discovery by allowing detection of secreted molecules or imaging readouts that correlate with CNS pathology. As demonstrated by Barolo et al. (2024), the integration of the tau fluorescent probe BT1 within ferritin nanocages allowed for sensitive detection of intracellular tau aggregates in live iPSC-derived retinal neurons. These biosensing approaches could bridge *in vitro* phenotypes with clinical diagnostics.

The realm of precision medicine also benefits from these models. By capturing patient-specific genotypes, iPSC systems allow exploration of rare mutations, variable disease trajectories, and heterogeneous drug responses. Use of isogenic control lines further isolates the effects of specific variants, while large iPSC cohorts support population-based modeling. Collectively, these applications underscore the transformative potential of iPSC-derived retinal models in accelerating therapeutic discovery, biomarker identification, and personalized approaches to neurodegenerative disease.

**Limitations and challenges:** Despite their promise, iPSC-derived retinal models face several critical challenges. Many systems exhibit developmental immaturity, limiting their relevance for age-associated diseases. Aging protocols such as telomerase inhibition or progerin overexpression remain poorly standardized. Additionally, current models often lack the microenvironmental complexity of the *in vivo* retina, including microglia, vasculature, and immune signaling. This restricts their utility for modeling neuroinflammation or neurovascular interactions. Furthermore, differentiation protocols may suffer from variability across labs, affecting reproducibility. Finally, the scalability of these systems remains a barrier to widespread use in drug discovery or industrial pipelines. Addressing these limitations will be essential to fully harness the translational power of iPSC-derived retinal models and ensure their robust application in both research and clinical contexts.

**Future directions — engineering and clinical integration:** The future of retinal disease modeling lies in combining bioengineering, high-dimensional omics, and real-world clinical data. Ferraro et al. (2023) recently introduced the bio-imaging model eye, a modular optical system that mimics the geometry and optics of the human eye. This platform enables fluorescence-based imaging of iPSC-derived retinal tissue under realistic conditions, facilitating biomarker validation. Retina-on-a-chip systems, incorporating microfluidics, immune components, and vascularized scaffolds, are also under development to simulate perfused, multicellular environments.

In parallel, single-cell transcriptomics and spatial proteomics now allow deep profiling of retinal organoids and RPE cultures, revealing cell-type–specific responses to Aβ and tau. These data sets may uncover early biomarkers or therapeutic targets. Moreover, live-cell imaging with genetically encoded sensors — for calcium, mitochondrial potential, or tau phosphorylation — enables dynamic monitoring of neurodegeneration *in vitro*. Perhaps most critically, aligning retinal phenotypes with patient-derived optical coherence tomography or fundus images could validate iPSC-derived systems for clinical translation.

iPSC-derived retinal models are transforming our understanding of tauopathy and retinal degeneration. Their human relevance, patient specificity, and scalability across platforms make them indispensable tools for preclinical research, therapeutic discovery, and precision medicine. As engineering advances and clinical integration deepen, these systems are poised to become central to the translational pipeline — unlocking new diagnostic and therapeutic frontiers for blinding and neurodegenerative diseases alike.

In addition to their role as accessible neural tissue, the retina and its derived models uniquely enable longitudinal studies of disease progression. Repeated imaging and functional testing can be applied non-invasively, allowing correlation of structural changes with molecular biomarkers over time. This is particularly valuable in preclinical stages, where early intervention may alter disease trajectory. Leveraging this capacity, retinal organoids can be periodically assessed for tau aggregation, synaptic decline, or inflammatory shifts in response to environmental stimuli or candidate therapeutics.

Furthermore, the use of CRISPR/Cas9 gene-editing in iPSC-derived retinal cells allows for precise manipulation of disease-associated mutations. For instance, correction of the MAPT IVS10+16 mutation in retinal neurons could serve as a platform for testing allele-specific therapies. Similarly, insertion of apolipoprotein E ε4 alleles into isogenic lines facilitates exploration of lipid dysregulation and its retinal consequences, building a link between AD genetic risk and retinal degeneration.

An important consideration in translational modeling is the fidelity of neurovascular interactions. The retina is highly vascularized, and its degeneration often involves blood-retina barrier breakdown, microvascular leakage, and ischemia. However, current retinal organoid systems lack integrated vasculature. Efforts to include endothelial precursors or to transplant organoids into vascularized scaffolds (e.g., in the chorioallantoic membrane or ocular chamber) are underway. These approaches aim to replicate hypoxia-driven mechanisms and improve drug delivery models.

Recent research also underscores the importance of mitochondrial dysfunction in retinal neurodegeneration. As highly metabolically active tissue, retinal neurons and RPEs are exquisitely sensitive to deficits in mitochondrial dynamics, oxidative phosphorylation, and calcium buffering. iPSC-derived models allow detailed interrogation of mitochondrial stress responses using live-cell reporters such as mitochondrial superoxide, Tetramethylrhodamine ethyl ester, or genetically encoded sensors. Targeting mitochondrial resilience may emerge as a cross-disease strategy to counteract neurodegeneration.

Clinically, these systems may also help bridge the diagnostic gap in patients with ambiguous cognitive profiles but early visual symptoms. For example, differentiating primary ocular conditions from CNS-driven visual decline (e.g., posterior cortical atrophy) remains challenging. Retinal imaging integrated with molecular markers from iPSC-derived cultures may enable earlier differential diagnosis, particularly in atypical or mixed-dementia phenotypes.

In summary, iPSC-derived retinal models not only reproduce key elements of neurodegenerative pathology but also offer a biologically rich and clinically relevant platform. By incorporating omics, engineering, and clinical correlates, these systems are well positioned to catalyze a new era of personalized diagnostics and therapeutics in neurology and ophthalmology.

Another promising frontier involves the integration of immune components into retinal models. Microglia, the resident immune cells of the CNS, play a crucial role in synaptic pruning, debris clearance, and neuroinflammation. Their absence in conventional retinal organoids limits the ability to model inflammatory cascades implicated in tauopathy. Recent protocols have started to incorporate iPSC-derived microglia into organoid systems, demonstrating interactions with photoreceptors and inner retinal neurons. This advance enables studies on microglial-mediated tau clearance, phagocytosis, and secretion of pro- or anti-inflammatory cytokines, which are essential for modeling the immunopathology of neurodegenerative disease.

Electrophysiological profiling of iPSC-derived retinal neurons and organoids adds another layer of functional validation. Multi-electrode arrays, calcium imaging, and patch-clamp recordings enable real-time assessment of synaptic transmission, action potential dynamics, and network connectivity. These technologies can be used to quantify tau-induced synaptic silencing or amyloid-driven hyperexcitability, providing readouts for drug screening and mechanistic exploration. HD-multi-electrode array platforms, such as BioCAM from 3Brain (Pfäffikon, Switzerland), allow spatial mapping of retinal activity across layers, adding precision to phenotype characterization.

To further enhance clinical relevance, iPSC-derived retinal systems may be personalized by integrating patient-derived serum or cerebrospinal fluid. This strategy allows the modeling of systemic influences on retinal health, such as pro-inflammatory cytokines, circulating autoantibodies, or dysregulated lipid metabolites. Combined with live-cell imaging, this approach could help identify biomarkers of systemic exposure and bridge the gap between molecular pathology and peripheral sampling in clinical practice.

It is worth noting that iPSC-derived 2D and 3D retinal models have shown significant promise for clinical applications, particularly in the treatment of retinal degenerative diseases. These models are actively being investigated for their potential to restore vision by replacing damaged retinal cells. Both preclinical studies and clinical trials have demonstrated the feasibility of transplanting iPSC-derived retinal cells, highlighting their capacity to integrate with host tissues and restore partial visual function. For instance, iPSC-derived retinal sheets transplanted into animal models such as pigs and monkeys have successfully integrated with host retinas and formed new photoreceptor layers (Uyama et al., 2022). A clinical trial in Japan (jRCTa050200027) has further demonstrated the translational potential of this approach, using iPSC-derived retinas to treat patients with retinitis pigmentosa (Uyama et al., 2022). Moreover, combining iPSC technology with emerging tools such as 3D bioprinting and microfluidic platforms (Salaris et al., 2019) may further enhance the precision, maturation, and therapeutic efficacy of retinal models, thereby facilitating their clinical translation.

However, managing immune rejection remains a critical challenge for the success of iPSC-derived retinal transplants. While studies have shown that major histocompatibility complex-matched grafts can significantly reduce immune responses, additional research is needed to optimize immunological compatibility and improve long-term transplant outcomes (Uyama et al., 2022). Thus, although iPSC-derived retinal models hold substantial therapeutic promise, overcoming hurdles such as immune rejection and advancing enabling technologies will be essential. Continued research and clinical development are crucial to fully unlock the potential of iPSC-derived retinal therapies for retinal degenerative diseases (Cho et al., 2019).

Importantly, these advanced systems also support ethical frameworks for reduction of animal usage in biomedical research. By capturing human-specific disease phenotypes and drug responses, iPSC-derived retinal models offer a powerful alternative to rodent models, which often fail to recapitulate key aspects of human tauopathies. Moreover, organoid platforms are highly adaptable for studying rare mutations or mixed pathologies that are difficult to model *in vivo*, contributing to a more inclusive and precise understanding of disease heterogeneity. As summarized in **Figure 1**, iPSC-derived retinal models are poised to bridge mechanistic insights with clinical translation in tauopathies and retinal neurodegeneration.

*This work was supported by D-Tails-IIT Joint Lab; Progetto ECS 0000024 Rome Technopole, Grant/Award Number: CUP B83C22002820006; PNRR Missione 4 Componente 2 Investimento 1.5, Italian; Ministry of Health (MoH) Alternative Methods to Animal Testing Grant 2023, Grant/Award Number: NEURO-3R; Regione Lazio, Grant/Award Number: A0112E0073; Italian Ministry of University and Research (MUR), Grant/Award Numbers: FISA-2023-00045, CUPB83D23001150001, PRIN2022 CUP2022CFP7RF (all to SDA).*

*No conflicts of interest exist between D-Tails Research srl BC and the publication of this work.*
